# A Taylor-Made Design of Phenoxyfuranone-Type Strigolactone Mimic

**DOI:** 10.3389/fpls.2017.00936

**Published:** 2017-06-20

**Authors:** Kosuke Fukui, Daichi Yamagami, Shinsaku Ito, Tadao Asami

**Affiliations:** ^1^Department of Applied Biological Chemistry, Graduate School of Agricultural and Life Sciences, The University of TokyoTokyo, Japan; ^2^Bioactive Natural Products Research Group, King Abdulaziz UniversityJeddah, Saudi Arabia; ^3^Biochemistry Department, Faculty of Science, King Abdulaziz UniversityJeddah, Saudi Arabia

**Keywords:** strigolactone, debranone, *Striga hermonthica*, suicidal germination, branching

## Abstract

Strigolactones are a class of plant hormones that inhibit axillary bud outgrowth and are released from plant roots to act as a rhizosphere communication signal. The Orobanchaceae parasitic plant *Striga hermonthica* perceives strigolactone as its germination signal, indicating host presence. After germination, the *Striga* plant parasitises the host plant and suppresses host growth by draining photosynthetic products, water and other essential nutrients. Because of this way of life, this parasite threatens crop production in sub-Saharan Africa with infestation in crop fields and crop devastation. Crop protection in such areas is among the most concerning problems to be dealt with as immediately as possible. With respect to crop protection from *Striga*, many strigolactone agonists have been developed and used in research to reveal *Striga* biology, and have contributed to development of crop protection methods. However, an effective method has yet to be found. In a previous study, we reported debranones as a group of strigolactone mimics that inhibit axillary buds outgrowth with moderate stimulation activity for *Striga* germination. Debranones would be accessible because they are simply synthesized from commercially available phenols and bromo butenolide. Taking this advantage of debranones for *Striga* research, we tried to find the debranones stimulating *Striga* seed germination. To modulate functional selectivity and to enhance germination inducing activity of debranones, we studied structure–activity relationships. We investigated effects of substituent position and functional group on debranone activity and selectivity as a strigolactone mimic. As a result, we improved stimulation activity of debranones for *Striga* seed germination by chemical modification, and demonstrated the pharmacophore of debranones for selective modulation of distinct strigolactone responses.

## Introduction

**FIGURE 1:**
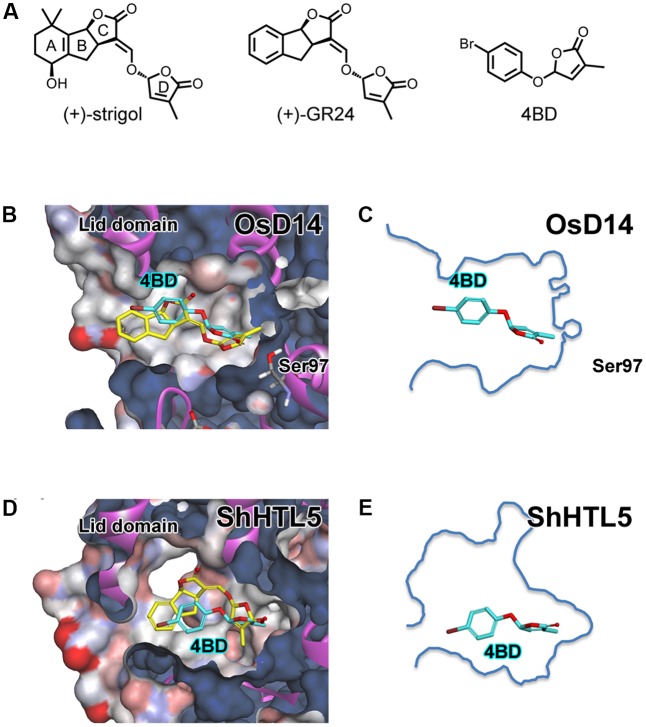
Structure of strigolactones and docking simulation of 4BD with OsD14 and ShHTL5. **(A)** (+)-strigol : a natural SL, (+)-GR24 : a synthetic SL analog and 4BD: an SL mimic. **(B)** Overlay structure of docked (*R*)-4BD on co-crystallized (+)-GR24 with OsD14 and OsD14 solvent surface. **(C)** (*R*)-4BD with outline of the OsD14 SL binding pocket. **(D)** Overlay structure of docked (*R*)-4BD and docked (+)-GR24 with ShHTL5 and ShHTL5 solvent surface. **(E)** (*R*)-4BD with outline of the ShHTL5 SL binding pocket. **(B,D)** Yellow stick indicates (+)-GR24 co-crystallized with OsD14 or docked with ShHTL5, respectively. **(B–E)** Cyan stick indicates (*R*)-4BD docked with the OsD14 or ShHTL5, respectively. All molecular docking simulation was performed using AutoDock Vina.

Global food security is among the most considerable issues in the world, since the increase in population has become a serious problem in the developing countries. In sub-Saharan Africa, a type of root parasitic plant, *Striga*, threatens food security by parasitising crop plants. *Striga* infection critically suppresses growth and development of host plants by draining carbohydrates and other essential nutrients from host plants. Moreover, *Striga* plants produce about one hundred thousand extremely small seeds that cannot be picked up by hand and that can remain dormant in the soil for more than 10 years while maintaining their germination ability (Joel et al., 1995, 2007). Therefore, the field would be forced to become a seed bank of *Striga* if an infestation occurred. As a result, *Striga* infestation can spread so effectively that *Striga*-induced crop loss was estimated to be more than $7 billion U.S. dollars annually across the African continent (Ejeta, 2007). In this context, it is worth trying to develop a successful method to prevent crops from *Striga* infection. As an obligate parasite, *Striga* cannot live without a host–parasite connection. *Striga* plants sense small molecules known as strigolactones (SLs) as germination signals, indicating host presence. By exploiting this germination system triggered by SLs, germination control may be an effective method against *Striga* infestation. To stimulate *Striga* seed germination, artificial SL analogs were developed based on the structures of natural SLs through structure–activity relationship studies (Johnson et al., 1981; Nefkens et al., 1997). For instance, the most potent SL analog, GR24 (**Figure 1A**), was developed as a Strigol analog. Use of these synthetic compounds has advanced research on *Striga* germination, and has contributed to development of crop protection methods against *Striga*. For instance, it was revealed that such agonist could be useful for removal of stored *Striga* seeds in the field by chemical stimulation of non-host germination, called suicidal germination (Zwanenburg et al., 2009; Samejima et al., 2016). However, a reasonably effective solution has yet to be found.

In the 2000s, SLs were rediscovered as a novel class of plant hormone that inhibit axillary shoot growth and act as a rhizosphere communication signal that stimulates hyphal branching of arbuscular mycorrhizal fungi (Akiyama et al., 2005; Gomez-Roldan et al., 2008; Umehara et al., 2008). The common structure of natural SLs consists of three fused rings (ABC ring) containing one lactone ring (C ring) and another lactone ring (D ring) connected to the C ring via an enol ether moiety (**Figure 1A**). In plants, SLs are biosynthesised from all-*trans*-β-carotene through several steps of enzymatic reactions involving one isomerase, two carotenoid cleavage dioxygenases and cytochrome P450(s) (Alder et al., 2012; Abe et al., 2014; Cardoso et al., 2014; Seto et al., 2014; Zhang et al., 2014). A portion of biosynthesised SLs is exuded from the roots to the soil to elicit a symbiotic interaction with arbuscular mycorrhizal fungi, whereas internal SLs, working within the plant body, regulate downstream gene expression to control plant architecture and physiological responses via SL receptors. SL receptors of model plants have been identified recently as α/β hydrolase proteins DAD2, OsD14, AtD14 and RMS3 in petunia, rice, *Arabidopsis thaliana* and pea, respectively (Hamiaux et al., 2012; Waters et al., 2012; Nakamura et al., 2013; Kameoka et al., 2016). Furthermore, SL perception mechanisms were recently proposed using X-ray crystallography and LC-MS/MS analysis (Jiang et al., 2013; Zhou et al., 2013; de Saint Germain et al., 2016; Yao et al., 2016). The D14 protein receives SL and enzymatically cleaves the enol ether bond to form a new bond with catalytic residue, which then activates D14 binding to a D3/MAX2 F-box protein to form the SCF^D3/MAX2^ ubiquitin E3 ligase and recruits D53/SMXLs repressor proteins for ubiquitination in rice and *Arabidopsis*, respectively. Ubiquitinated repressors are degraded by the 26S proteasome system, and then SL signaling is up-regulated. This perception mechanism seems to be applicable to SL perception by *Striga*. In *Arabidopsis*, the *D14* paralog *KAI2*/*HTL* is involved in seed germination, and KAI2/HTL signal transduction shares MAX2 with D14 signaling (Nelson et al., 2011). *AtKAI2*/*HTL* orthologs in *Striga hermonthica*, *ShKAI2s*/*HTLs*, have been identified and tested to demonstrate their ability to complement *AtKAI2*/*HTL* function in *Arabidopsis kai2*/*htl* mutants (Conn et al., 2015; Toh et al., 2015). These findings might contribute to the elucidation of host recognition mechanism by *Striga* and to the development of advanced crop protection methods from *Striga* infestation.

In the previous research, we developed a set of simple and accessible SL agonists that potently inhibit shoot branching in plants (Fukui et al., 2011, 2013). However, these compounds did not potently stimulate *Striga* seed germination, the other SL biological function. Until then, no SL agonist distinctively stimulates different SL responses. We regarded our compounds as function-specific SL agonists, and named them debranones because of their potent de-branching activity. Such alternative agonists have provided significant findings in plant hormone research. For instance, pyrabactin, an abscisic acid (ABA) analog, and coronatine, a jasmonoyl isoleucine (JA-Ile) analog, contributed to identification of ABA receptors and JA-Ile receptors, respectively (Park et al., 2009; Yan et al., 2009). Likewise, our representative compound 4-bromo debranone (4BD; **Figure 1A**) was used in research to unveil molecular mechanisms of SL perception by D14 (Yao et al., 2016). These contributions should be provided mostly by availability of debranones. Each research group obtained debranones by simple reaction of commercially available phenol with halogenated butenolide in their own laboratory. Because its synthetic reaction is easily scalable, debranones could be used in large scale at a lower cost than other SL agonist such as GR24. We counted on such potential of debranones and attempted to develop new debranone-type substances applicable to *Striga* research. In this study, we aimed to improve the SL-like activity of debranones for seed germination of root parasitic plants using a structure–activity relationship study and rational chemical design based on the structure of the ShHTL5 SL binding pocket.

## Results

For rational design of subsequent debranones, we simulated a 4BD (**Figure 1A**)–OsD14 binding assay using AutoDock Vina (Trott and Olson, 2010; **Figures 1B,D**). To confirm the reliability of this simulation, we applied the docking calculation to GR24 with OsD14, and compared the docked pose of GR24 with the conformation of GR24 in the co-crystal of GR24–OsD14 (Supplementary Figure S1). This simulation result demonstrated that docked pose of GR24 was sufficiently overlapped the form of GR24 in the co-crystal of GR24–OsD14. Then, we applied the docking calculation to our compound 4BD. According to the proposed SL perception mechanism, the enzymatic SL hydrolysis reaction by D14 triggers activation of itself to interact with the SCF^D3^ complex (Yao et al., 2016). Thus, SLs should be anchored near the Ser-His-Asp of the catalytic residue located on the bottom of the D14 SL binding pocket. In our simulation result, docked 4BD (cyan stick) was anchored near the catalytic residue, and the molecular conformation of docked 4BD considerably overlapped with GR24 (yellow stick), whose structure is obtained from co-crystals of OsD14 and GR24 (**Figure 1B**). Next, we ran a similar calculation using ShHTL5 of the AtKAI2/HTL ortholog, which is among the candidates for SL receptor in *S. hermonthica*, as a substitute for OsD14. The simulation result of ShHTL5 docked with GR24 (yellow stick) indicated GR24 could fit in the SL binding pocket of ShHTL5 as well as the result of OsD14 (**Figure 1D**). On the other hand, docked 4BD (cyan stick) was placed in the SL binding pocket of ShHTL5 in much the same fashion as the results of OsD14, although 4BD does not fill the cavity space of ShHTL5 (**Figures 1C–E**). Comparing 4BD with GR24, a 4-substituted phenyl ring is obviously smaller than tricyclic lactone. Moreover, it seems that a mono-substituted phenyl ring could rotate flexibly in the pocket due to the lack of steric hindrance and of functional groups to elicit ligand–receptor interaction. These observations implied that low affinity of 4BD to ShHTL receptors might result in the moderate activity for *Striga* germination. Thus, we hypothesized that structural modification on the phenyl ring might improve debranone affinity to SL receptors for *Striga* germination and enhance biological activity of those compounds. Our previous study revealed that the position of functional group on phenyl ring dramatically affected both branching inhibitory activity for rice and germination inducible activity for *Striga* seed (Takahashi et al., 2016). Therefore, we synthesized di-substituted debranones and tested their multiple SL-like activities.

**FIGURE 2:**
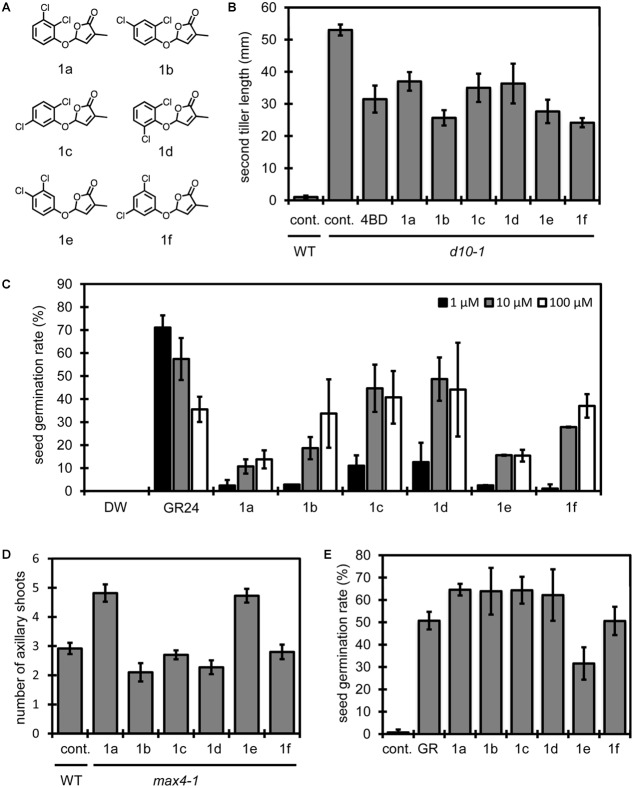
Structure of dichloro-debranones and assay results. **(A)** Structures of dichloro-debranones. **(B)** Results of tillering inhibition assay of rice. Each bar indicates average length of second tiller of six rice seedlings 16 days after germination. Error bar means SE. Each seedling was grown in hydroponic culture with indicated compounds at 10 nM concentration or DMSO for 7 days before measurement. **(C)** Results of germination stimulation assay for *Striga hermonthica*. Each bar indicates average germination rate of 6 sets of seeds. About 40 seeds were treated with each compound at indicated concentration for 2 days before counting in each set. Error bar means SD. All seeds were conditioned for 14 days before chemical treatment. **(D)** Results of branching inhibition assay of *Arabidopsis*. Each bar indicates average number of rosette shoots per *Arabidopsis* plant growing 42 days after germination. *n* = 10–12 number of individual plants, error bar means SE. Each plant was grown in hydroponic culture media with indicated compounds at 1 μM concentration or DMSO as a control for 21 days before measurement (cont., control). **(E)** Results of germination stimulation assay for *Arabidopsis* seed under high temperature conditions. Each bar indicates average germination rate of three sets of seeds. About 50 seeds were treated with indicated compound at 10 μM in each set. Error bar means SD. All seeds were imbibed in distilled water with or without chemicals for 2 days at 30°C before counting (GR, GR24).

Next, we synthesized a set of compounds derived from dichlorophenols to investigate relationships between substituent position and SL activity in all combinations with the two chlorine atoms (**Figure 2A**). We evaluated the activities of these compounds using the following four types of biological assays: the first is a tillering inhibition assay for second tiller buds of SL-deficient rice mutant *d10*-*1* (Arite et al., 2007; **Figure 2B**); the second is a germination stimulation assay for *S. hermonthica* seed (**Figure 2C**); the third is a branching inhibition assay for rosette shoots of *Arabidopsis* SL-deficient mutant *max4-1* (Sorefan et al., 2003; **Figure 2D**); and the last is a germination stimulation assay for *Arabidopsis* under high temperature conditions (Toh et al., 2012; **Figure 2E**). Rice second tiller lengths shown in **Figure 2B** implied us that compounds **1b**, **1e** and **1f** inhibit tiller bud outgrowth more effectively than 4BD at 10 nM concentration. Same trend was observed in another assay at 1 nM concentration (Supplementary Figure S2). Compounds **1a**, **1c** and **1d** also exerted an inhibitory effect, though their effectiveness seemed less than that of **1b** and **1f**. On the other hand, *Striga* germination rates shown in **Figure 2C** indicate that stimulation effects of compounds **1c** and **1d** are stronger than others in a set of a dichloro-debranone series. Next, we applied those compounds to other two types of biological assays using *Arabidopsis* (**Figures 2D,E**). **Figure 2D** shows the number of rosette branches with each dichloro-debranone treatment, and **Figure 2E** shows germination rates with each chemical treatment under high temperature conditions. These results indicate that compound **1a** has weak inhibitory activity on *Arabidopsis* rosette shoots and that compound **1e** is inactive in both assay systems. The overall results above demonstrate that the structure–activity relationships of the tested compounds are different in these four assays. That is, different combinations of substituents on the phenyl ring are required to exert each SL function *in vivo*. This fact implies the possibility that we could design chemicals to activate the selected SL function.

**FIGURE 3:**
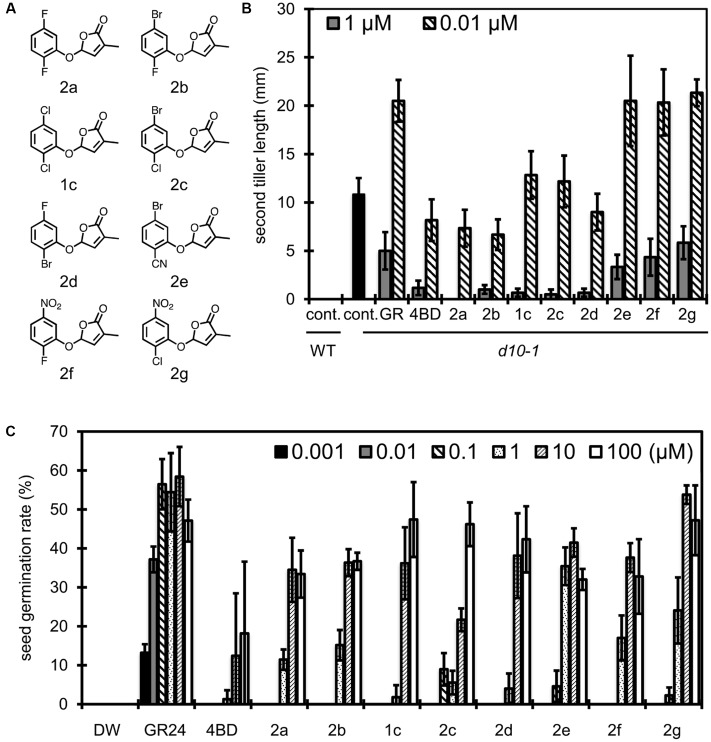
Structure of 2,5-disubstituted debranones and assay results. **(A)** Structures of synthesized 2,5-disubstituted debranones. **(B)** Results of tillering inhibition assay of rice. Each bar indicates average length of second tiller of six rice seedlings 16 days after germination. Error bar means SE. Each seedling was grown in hydroponic culture with each compound at the indicated concentration for 7 days before measurement (cont., control; GR, GR24). **(C)** Results of germination stimulation assay for *Striga hermonthica*. Each bar indicates average germination rate of three sets of seeds. About 40 seeds were treated with each compound at the indicated concentration for 2 days before counting in each set. Error bar means SD. All seeds were conditioned for 14 days before chemical treatment.

**FIGURE 4:**
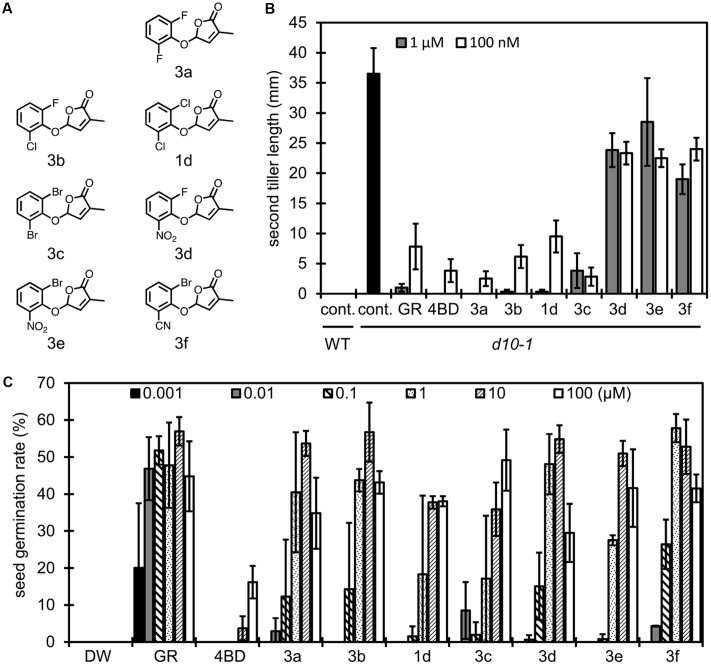
Structure of 2,6-disubstituted debranones and assay results. **(A)** Structures of synthesized 2,6-disubstituted debranones. **(B)** Results of tillering inhibition assay of rice. Each bar indicates average length of second tiller of six rice seedlings 16 days after germination. Error bar means SE. Each seedling was grown in hydroponic culture with each compound at the indicated concentration for 7 days before measurement (cont., control; GR, GR24). **(C)** Results of germination stimulation assay for *Striga hermonthica*. Each bar indicates average germination rate of three sets of seeds. About 40 seeds were treated with each compound at the indicated concentration for 2 days before counting in each set. Error bar means SD. All seeds were conditioned for 14 days before chemical treatment.

Based on the studies of relationships between substituent position and SL activities of debranones as described above, we selected 2,5-dichlorodebranone and 2,6-dichlorodebranone as lead compounds for *Striga* germination induction. To improve the efficacy of debranones for SL-induced *Striga* germination, we investigated the effects of individual functional groups. In our previous study, an electron donating group did not enhance SL like activity of compounds for both rice tillering inhibition and *Striga* germination induction (Fukui et al., 2011). Based on the previous observation, we functionalized 2,5- and 2,6-disubstituted debranones with electron withdrawing groups, such as a halogen group, nitro group and cyano group. We evaluated the activities of derivatives on two types of assay systems using rice and *Striga* in the same manner as described above. Then, to find more selective debranones against for SL-induced *Striga* germination, we compared the results of those two assays. 2,5-Disubstituted debranones (compounds **1c** and **2a–g**) shown in **Figure 3A** and 2,6-disubstituted debranones (compounds **1d** and **3a–f**) shown in **Figure 4A** were easily obtained from one step reaction between commercially available phenols and bromobutenolide with good yield (more than 60%). Regarding inhibition effects of 2,5-disubstituted debranones on second tiller bud outgrowth of rice *d10-1* (**Figure 3B**), **1c** and **2a–d**, whose compounds are functionalised by the halogen substituent, the second tiller bud outgrowth was inhibited almost perfectly at 1 μM, but compounds **2e–g**, which are functionalised with a strong electron withdrawing group, did not inhibit tiller bud outgrowth completely at this concentration. At 0.01 μM, **2a**, **2b** and **2d** inhibited the second tiller bud outgrowth at the same level as 4BD. On the other hand, in terms of the stimulation effects of compounds **1c** and **2a–g** on *Striga* seed germination (**Figure 3C**), **2e–g** clearly induced *Striga* germination at concentrations greater than 1 μM, whereas compounds **1c**, **2c** and **2d** induced less germination than **2e–g** in this assay. These results showed opposite trend to the results obtained in the rice tillering inhibition assay, in which compounds **1c**, **2c** and **2d** showed good inhibitory activities.

In the same way, we evaluated the effects of functional groups in 2,6-disubstituted debranones (compounds **1d** and **3a–h**). Tillering inhibition activities of **1d** and **3a–c** were comparable to those of 4BD and GR24 at 100 nM (**Figure 4B**), whereas **3d–f**, functionalised with a strong electron withdrawing group, slightly inhibited tillering even at 1 μM. In contrast, **3d–f** showed potent stimulation effects on *Striga* seed germination. In addition to the results shown in **Figure 3C**, halogenated-type compounds were relatively weak with respect to germination stimulation for *Striga*, with only compounds **3a** and **3b**, functionalised with fluoro groups, showing significant effects on *Striga* seed germination at the same level as **3d–f**. To summarize the above results, it seemed that **3f** was the most effective compound for inducing *Striga* seed germination among all debranones. Furthermore, **3f** can selectively stimulate seed germination of *Striga* without inhibition of rice tiller bud outgrowth. From the standpoint of functional selectivity, a halogen group substituent enhanced branching inhibition effects in rice, and a strong electron withdrawing group enhanced stimulation effects on *Striga* germination. These effects of functional groups seem to occur regardless of their substitution position.

**FIGURE 5:**
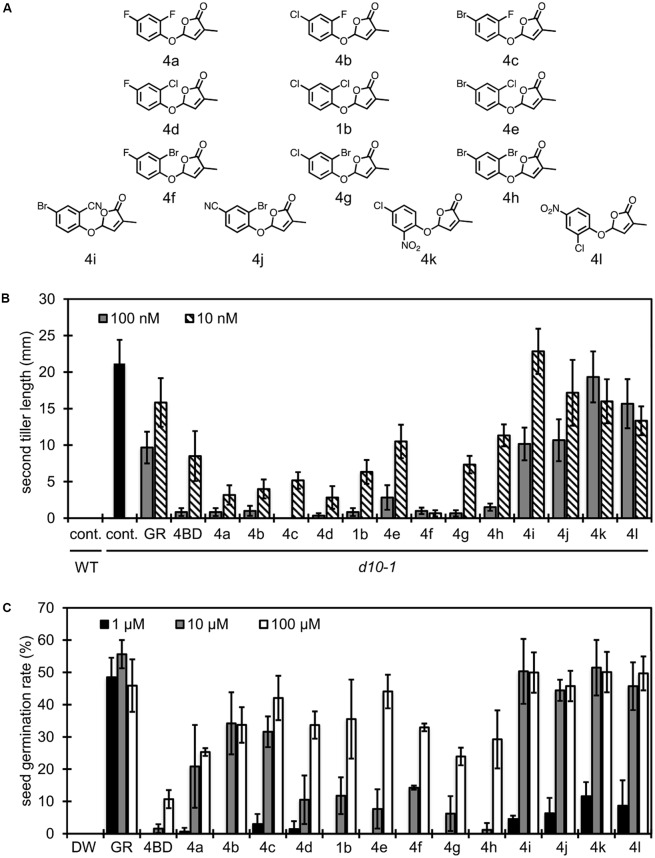
Structure of 2,4-disubstituted debranones and assay results. **(A)** Structures of synthesized 2,4-disubstituted debranones. **(B)** Results of tillering inhibition assay of rice. Each bar indicates average length of second tiller of six rice seedlings 16 days after germination. Error bar means SE. Each seedling was grown in hydroponic culture with each compound at the indicated concentration for 7 days before measurement (cont., control; GR, GR24). **(C)** Results of germination stimulation assay for *Striga hermonthica*. Each bar indicates average germination rate of three sets of seeds. About 40 seeds were treated with each compound at the indicated concentration for 2 days before counting in each set. Error bar means SD. All seeds were conditioned for 14 days before chemical treatment.

Based on the studies of substituent effects for debranones as demonstrated above, functional selectivity of compounds seems to be varied according to position of substituents on the phenyl ring as well as electronic and steric characteristics of the functional group. Thus, we tried to redesign compounds that are suitable only for branching inhibition in rice. As shown in **Figure 5A**, 2,4-disubstituted debranones were synthesized for this purpose. When comparing results of the rice tillering inhibition assay with those of the *Striga* germination stimulation assay, substituent effects were clearly observed (**Figure 5B**). Halogen substitution strengthened the tillering inhibitory activity, although fluorine atom attached in the 2-position enhanced the stimulation activity for *Striga* germination. In contrast, substitution with a strong electron withdrawing group, such as a nitro or cyano group, enhanced the stimulation activity for *Striga* germination, but the substitution attenuated the inhibitory effects on rice tillering, regardless of the substituted position. As a result, compound **4f** was the most active compound in the rice tillering inhibition assay (**Figure 5B**), while it did not stimulate seed germination of *Striga* at lower concentration than 1 μM (**Figure 5C**). **4f** showed functional selectivity at the same level as 4BD.

## Discussion

The results of the 4BD–ShHTL5 docking simulation *in silico* showed us that 4BD did not fit into SL binding pocket of ShHTL5 well. SL binding pocket of ShHTL5 seems larger than that of OsD14. Therefore, based on the hypothesis, we synthesized di-substituted debranones to fit them to SL binding pocket of ShHTL receptors well. Indeed, 4BD is less effective than any other 2,4-, 2,5- or 2,6-disubstituted debranone derivative in *Striga* assays (**Figures 3C**, **4C**, **5C**). Those results at least supported that two substituents on phenyl ring contribute to stimulation activity of debranones for *Striga* seed germination. In addition, the target selectivity of the compounds could be affected by the combination of substituent positions within a dichloro-debranone series (**Figure 2C**). We supposed that such effect of two substitutions would be attributed by the differences of ligand–receptor interaction from mono-substitution. In this study, it was demonstrated in several assays using plants that the second substituent on phenyl ring plays a critical role not only for activity but also for selectivity of debranones. Substituted functional groups of debranones also affect intra-molecular polarity and acidity of phenols, which can be converted to corresponding debranones. A previous report suggested that there should be relationships between the pKa value of phenols and stimulation activity of corresponding debranones for *Striga* germination (Tsuchiya et al., 2015). In that paper, the mono-substituted debranones, which were derived from phenols with the lower pKa values than non-substituted phenols, were more active than non-substituted debranone in stimulating *Striga* seed germination. Generally, in chemistry, conjugated bases of highly acidic compounds are good leaving groups. In other words, low pKa acids should be good functional groups behaving as good leaving groups. This general rule suggests that the debranones derived from the low pKa phenols in this report should be readily hydrolysed by SL receptors to eliminate the corresponding phenols. According to the SL perception mechanism reported recently, activation of SL signaling is triggered by conformational change of D14 following covalent bond formation between SL hydrolysate and D14 (de Saint Germain et al., 2016; Yao et al., 2016). If the *Striga* SL receptors act in the same manner as the D14 working model, it is reasonable to assume that the debranones, derived from the low pKa phenols, would be potent for stimulating *Striga* seed germination. Therefore, we compared pKa values of all phenols with the activities of corresponding debranones in this study (Supplementary Table S1). As an overview of this table, as expected, active debranones in the *Striga* assay are derived from phenols functionalised with strong electron withdrawing groups (cyano group and nitro group). However, intriguingly, some debranones, such as **1a** and **1c**, which are derived from different phenols with the same pKa, showed distinctive active profile in SL assays. These results suggest that the germination-inducing activity of compounds would be determined by both ligand binding affinity to SL receptors and susceptibility to hydrolysis by the receptors.

At the beginning of this study, we aimed to develop new debranones available for *Striga* researches that stimulate seed germination of *Striga* more actively than existing debranones. As expected, we achieved this goal through a structure–activity relationship study based on the hypothesis described above. Moreover, several debranones, that can actively induce *Striga* germination, exhibited far less activity for tillering inhibition in rice than that of 4BD. The selectivity of these compounds was opposite to that of previously developed debranones: they were active in the inhibition of rice tillering but not active in the stimulation of *Striga* seed germination. The cause of this target selectivity of these compounds should be based on various biological factors, such as transport, metabolism and perception. Comparing the biological activities among the dichloro-debranone series, **1c** and **1d** were relatively active in the germination stimulation assay of *Striga* but relatively less effective than others in inhibiting rice tillering (**Figure 2**). This difference might not come from ligand–receptor interaction because both **1c** and **1d** showed high potency for inhibiting shoot branching of *Arabidopsis* (**Figure 2D**). Our data demonstrated that the introduction of strong electron withdrawing groups, such as a cyano group or nitro group, enhanced the effect of debranones on *Striga* but impaired their effects on rice. This difference could be attributed to the stability of compounds in culture media because the period of the rice tillering inhibition assay was longer than that of the *Striga* germination induction assay. Electron withdrawing groups would make debranone readily hydrolysable via D14 hydrolase, while they should make debranone hydrolytically degraded by water in culture media and the plant body. Here, we developed various debranone derivatives selective for distinct SL responses in rice, *Striga* and *Arabidopsis* plants. These target selective SL agonists have potential for *Striga* control and can become lead chemicals for development of new chemical tools, such as yoshimulactone (Tsuchiya et al., 2015).

## Materials and Methods

### Docking Simulation

We used OsD14 structural information extracted from the OsD14–GR24 co-crystal (PDB ID: 5DJ5) (Zhao et al., 2015). We used ShHTL5 structural information extracted from the crystal of ShHTL5 (PDB ID: 5CBK) (Toh et al., 2015). Molecular docking simulation was performed in AutoDock Vina via PyRx-0.8 software (Trott and Olson, 2010).

### Chemicals

#### Synthesis of GR24

GR24 was synthesized as described in the literature (Mangnus et al., 1992). We used racemic (±)-GR24 with the same relative stereochemistry as (±)-strigol in all assays of this study.

#### Synthesis of 5-Bromo-3-methyl-2(5*H*)-furanone

5-Bromo-3-methyl-2(5*H*)-furanone was synthesized using the following described procedure. N-Bromosuccinimide (10.65 g, 60 mmol) and 2,2′-azobis(isobutyronitrile) (0.20 g, 1.2 mmol) were added to a 200-mL round flask and suspended with 60 mL of carbon tetrachloride. 3-methyl-2(5*H*)-furanone (5 mL, 60 mmol) was added to this mixture and was then refluxed at 90°C for 4 h. After reaction mixture was cooled to room temperature, precipitates were removed by filtration. Resultant solution was evaporated *in vacuo*, and then purified by silica gel column chromatography (pale yellow oil, 9.95 g, 90% yield).

### General Procedure

#### Spectroscopy

Proton NMR spectra of all compounds were recorded with a JEOL ECA 500 II (500 MHz) spectrometer. The exact mass of all compounds was obtained using a Triple TOF^TM^ 5600 System (AB SCIEX). All data are listed in the Supplementary Data Sheet 1.

#### Synthesis of Di-substituted Debranones

All debranones were synthesized from commercially available phenols and 5-bromo-3-methyl-2(5*H*)-furanone. Compounds **1a**–**f**, **2b**, **2d**, **2e** and **4a**–**j** were synthesized using the following described method. An amount of 1.2 mmol of sodium hydride was added to a 20-mL round flask and suspended with 2 mL of dry THF. To this mixture, 1 mmol of substituted phenol dissolved into 2 mL of dry THF was slowly added. Then, 1 mmol of 5-bromo-3-methyl-2(5*H*)-furanone dissolved into 2 mL of dry THF was slowly added to the solution mixture. The reaction mixture was stirred for 2 h at room temperature and monitored by TLC (*n*-hexane:ethyl acetate = 4:1). The reaction mixture was quenched with 5 mL of water and then extracted twice with 5 mL of ethyl acetate. The extracted organic layer was combined and dried over anhydrous sodium sulfate, and the layer was then concentrated *in vacuo* and purified by silica gel column chromatography. Compounds **2a**, **2c**, **2f**, **2g**, **3a–d**, **3f**, **4k** and **4l** were synthesized using the following described method. An amount of 1.2 mmol of potassium carbonate was added to a 20-mL round flask and suspended with 4 mL of acetone. To this mixture, 1 mmol of substituted phenol dissolved into 2 mL of acetone was slowly added. Then, 1 mmol of 5-bromo-3-methyl-2(5*H*)-furanone dissolved into 2 mL of acetone was slowly added to the solution mixture. The reaction mixture was stirred for 2 h at room temperature and monitored by TLC (*n*-hexane:ethyl acetate = 4:1). Five milliliters of water were added to the reaction mixture and then extracted twice with 5 mL of ethyl acetate. The extracted organic layer was combined and dried over anhydrous sodium sulfate, and the layer was then concentrated *in vacuo* and purified by silica gel column chromatography. Compound **3e** was synthesized using the following described method. An amount of 1.2 mmol of potassium carbonate and 1 mmol of tetrabutylammonium bromide were added to a 20-mL round flask; the solution was then dissolved into 2 mL of dichloromethane and 3 mL of water. One millimole of 2-bromo-6-nitrophenol and 1 mmol of 5-bromo-3-methyl-2(5*H*)-furanone were then dissolved into 2 mL of dichloromethane and slowly added to the solution mixture. The reaction mixture was stirred for 8 h at room temperature and monitored by TLC (*n*-hexane:ethyl acetate = 4:1). Five milliliters of water were added to the reaction mixture and then extracted twice with 5 mL of ethyl acetate. The extracted organic layer was combined and dried over anhydrous sodium sulfate, and the layer was then concentrated *in vacuo* and purified by silica gel column chromatography.

### Plant Material and Assay Conditions

#### Germination Stimulation Assay of *Striga* Seed

In this assay, *S. hermonthica* seed was kindly provided by Professor A.G. Babiker at the Sudan University of Science and Technology. The seed was sterilized with 1% (v/v) sodium hypochlorite solution containing 0.01% (v/v) Tween-20 for 15 min and washed five times with sterilized DW on a clean bench. Seed was then incubated on moist glass fiber filter paper (∅ = 5 mm, approximately 50 seeds each) under dark, 30°C conditions for 14 days (conditioning). After conditioning, the seed was transferred to a 96-well titer plate with the filter paper and treated with a compound solution, whose concentration is indicated in the Figure. After the plate was incubated under dark, 30°C conditions for 48 h, the numbers of germinated seeds and non-germinated seeds were counted.

#### Tillering Inhibition Assay of Rice

In this assay, the *d10-1* mutant was used as an SL-deficient plant, and a wild-type plant (cv Shiokari) was used as a control. Rice seed was sterilized with a 2.5% (v/v) sodium hypochlorite solution containing 0.01% (v/v) Tween-20 for 25 min and washed five times with sterilized DW on a clean bench. Seed was then soaked in DW and incubated under dark, 25°C conditions for 2 days. Germinated seed was transferred to hydroponic culture media solidified with 0.65% (w/v) agar and allowed to grow under continuous light conditions at 25°C for 7 days. Rice seedlings were then transferred to 12 mL of hydroponic culture media with or without chemical treatment and grown under 16/8 h (light/dark) conditions at 25°C. These culture media were replaced after 4 days of cultivation. Seven days after transfer, the length of the second tiller was measured.

#### Branching Inhibition Assay of *Arabidopsis thaliana*

In this assay, the *max4-1* mutant was used as an SL-deficient plant, and a wild-type plant (Col-0) was used as a control. Assay conditions followed the same methods as described in the literature (Fukui et al., 2013).

#### Germination Stimulation Assay of *Arabidopsis thaliana* under High Temperature Conditions

Col-0 seed was used for this assay. Assay conditions followed the same methods as described in the literature (Toh et al., 2012).

## Conclusion

Debranones were firstly discovered as functional selective SL mimics, which inhibit axillary buds outgrowth while moderately stimulate *Striga* seed germination. In this study, we prepared various di-substituted debranones to discover a new debranone-type substance, which strongly stimulates *Striga* seed germination. Then, we obtained a compound **3f**, which showed the most potent activity among debranones for stimulating *Striga* seed germination. Through this research, we achieved to find how to modulate the functional selectivity of debranones on the basis of structure–activity relationship study. These results imply that debranones could be designed as Taylor-made SL mimics for various SL receptors. We anticipate that debranones will become useful chemical tools for developing novel methods for *Striga* control and for elucidating the differences in SL perception and signal transduction between plant hormonal function and function as a rhizosphere communication signal.

## Author Contributions

TA and KF conceived and designed the experiments. KF and DY performed the experiment with guidance of SI. TA and KF wrote the manuscript. All authors contributed to the discussion and approved the final manuscript.

## Conflict of Interest Statement

The authors declare that the research was conducted in the absence of any commercial or financial relationships that could be construed as a potential conflict of interest.

## Abbreviations

DMSO: dimethyl sulfoxide
DW: distilled water
SD: standard deviation
SE: standard error
THF: tetrahydrofuran
TLC: thin-layer chromatography
WT: wild type.
