# Supplementary material for: A Taylor-Made Design of Phenoxyfuranone-Type Strigolactone Mimic
Source: Front Plant Sci. 2017 Jun 20;8:936. doi: 10.3389/fpls.2017.00936 (PMC5477565; doi:10.3389/fpls.2017.00936)
Supplement: Supplementary file 1 [file Table_1.PDF]

Table S1. pKa values of phenols converted to debranones

Entry	phenol	2-position	3-position	4-position	5-position	6-position	pKa value
1a	2,3-dichlorophenol	Cl	Cl	H	H	H	7.53
1b	2,4-dichlorophenol	Cl	H	Cl	H	H	8.05
1c	2,5-dichlorophenol	Cl	H	H	Cl	H	7.53
1d	2,6-dichlorophenol	Cl	H	H	H	Cl	7.02
1e	3,4-dichlorophenol	H	Cl	Cl	H	H	8.56
1f	3,5-dichlorophenol	H	Cl	H	Cl	H	8.04
2a	2,5-difluorophenol	F	H	H	F	H	7.71
2b	2-fluoro-5-bromophenol	F	H	H	Br	H	7.74
2c	2-chloro-5-bromophenol	Cl	H	H	Br	H	7.53
2d	2-bromo-5-fluorophenol	Br	H	H	F	H	7.43
2e	2-cyano-5-bromophenol	CN	H	H	Br	H	6.20
2f	2-fluoro-5-nitrophenol	F	H	H	NO_2	H	6.95
2g	2-chloro-5-nitrophenol	Cl	H	H	NO_2	H	6.72
3a	2,6-difluorophenol	F	H	H	H	F	7.45
3b	2-fluoro-6-chlorophenol	F	H	H	H	Cl	7.23
3c	2,6-dibromophenol	Br	H	H	H	Br	6.89
3d	2-fluoro-6-nitrophenol	F	H	H	H	NO_2	5.67
3e	2-bromo-6-nitrophenol	Br	H	H	H	NO_2	5.36
3f	2-bromo-6-cyanophenol	Br	H	H	H	CN	5.63
4a	2,4-difluorophenol	F	H	F	H	H	8.72
4b	2-fluoro-4-chlorophenol	F	H	Cl	H	H	8.26
4c	2-fluoro-4-bromophenol	F	H	Br	H	H	8.14
4d	2-chloro-4-fluorophenol	Cl	H	F	H	H	8.50
4e	2-chloro-4-bromophenol	Cl	H	Br	H	H	7.92
4f	2-bromo-4-fluorophenol	Br	H	F	H	H	8.44
4g	2-bromo-4-chlorophenol	Br	H	Cl	H	H	7.98
4h	2,4-dibromophenol	Br	H	Br	H	H	7.86
4i	2-cyano-4-bromophenol	CN	H	Br	H	H	6.60
4j	2-bromo-4-cyanophenol	Br	H	CN	H	H	6.30
4k	2-nitro-4-chlorophenol	NO_2	H	Cl	H	H	6.32
4l	2-chloro-4-nitrophenol	Cl	H	NO_2	H	H	5.43
