# Supplementary material for: A Taylor-Made Design of Phenoxyfuranone-Type Strigolactone Mimic
Source: Front Plant Sci. 2017 Jun 20;8:936. doi: 10.3389/fpls.2017.00936 (PMC5477565; doi:10.3389/fpls.2017.00936)
Supplement: Supplementary file 2 [file Data_Sheet_1.DOCX]

**Spectra data of compounds**

**5-(2,3-Dichlorophenoxy)-3-methyl-2(5*H*)-furanone(1a)**

^1^H NMR (CDCl_3_) δ 2.03 (t, 3H, *J* = 1.5 Hz), 6.24 (t, 1H, *J* = 1.5 Hz), 7.07 (t, 1H, *J* = 1.5 Hz), 7.20 (t, 1H, *J* = 8.0 Hz), 7.25-7.28 (m, 2H), HRMS (ESI): Calcd For C_11_H_9_O_3_Cl_2_ : [M+H]^+^: 258.9923. Found: *m/z* 258.9928.

**5-(2,4-Dichlorophenoxy)-3-methylfuran-2(5*H*)-one(1b)**

^1^H NMR (CDCl_3_) δ 2.02 (t, 3H, *J* = 1.5 Hz), 6.21 (t, 1H, *J* = 1.5 Hz), 7.06 (t, 1H, *J* = 1.5 Hz), 7.22-7.29 (m, 2H), 7.41 (d, 1H, *J* = 2.5 Hz), HRMS (ESI): Calcd For C_11_H_9_O_3_Cl_2_ : [M+H]^+^: 258.9923. Found: *m/z* 258.9925.

**5-(2,5-Dichlorophenoxy)-3-methylfuran-2(5*H*)-one(1c)**

^1^H NMR (CDCl_3_) δ 2.04 (t, 3H, *J* = 1.5 Hz), 6.24 (d, 1H, *J* = 1.5 Hz), 7.06-7.08 (m, 2H), 7.33 (d, 1H, *J* = 8.5 Hz), 7.37 (d, 1H, *J* = 2.0 Hz), HRMS (ESI): Calcd For C_11_H_9_O_3_Cl_2_ : [M+H]^+^: 258.9923. Found: *m/z* 258.9932.

**5-(2,6-Dichlorophenoxy)-3-methylfuran-2(5*H*)-one (1d)**

^1^H NMR (CDCl_3_) δ 2.04 (t, 3H, *J* = 1.5 Hz), 6.31 (d, 1H, *J* = 1.0 Hz), 7.10 (t, 1H, *J* = 8.3), 7.14 (t, 1H, *J* = 1.8 Hz), 7.35 (d, 2H, *J* = 8.5 Hz), HRMS (ESI): Calcd For C_11_H_9_O_3_Cl_2_ : [M+H]^+^: 258.9923. Found: *m/z* 258.9923.

**5-(3,4-Dichlorophenoxy)-3-methylfuran-2(5*H*)-one (1e)**

^1^H NMR (CDCl_3_) δ 2.02 (t, 3H, *J* = 1.5 Hz), 6.23 (t, 1H, *J* = 1.3 Hz), 6.98 (t, 1H, *J* = 1.5 Hz), 7.00 (dd, 1H, *J* = 9.0, 3.0 Hz), 7.27 (d, 1H, *J* = 3.0 Hz), 7.39 (d, 1H, *J* = 9.0 Hz), HRMS (ESI): Calcd For C_11_H_9_O_3_Cl_2_ : [M+H]^+^: 258.9923. Found: *m/z* 258.9925.

**5-(3,5-Dichlorophenoxy)-3-methylfuran-2(5*H*)-one (1f)**

^1^H NMR (CDCl_3_) δ 2.03 (t, 3H, *J* = 1.5 Hz), 6.24 (d, 1H, *J* = 1.5 Hz), 6.97 (t, 1H, *J* = 1.8 Hz), 7.06 (d, 2H, *J* = 2.0 Hz), 7.12 (t, 1H, *J* = 2.0 Hz), HRMS (ESI): Calcd For C_11_H_9_O_3_Cl_2_ : [M+H]^+^: 258.9923. Found: *m/z* 258.9929.

**5-(2,5-Difluorophenoxy)-3-methylfuran-2(5*H*)-one (2a)**

^1^H NMR (CDCl_3_) δ 2.01 (d, 3H, *J* = 1.5 Hz), 6.25 (d, 1H, *J* = 1.0 Hz), 6.75-6.81 (m, 1H), 7.03 (t, 1H, *J* = 1.8 Hz), 7.04-7.10 (m, 2H), HRMS (ESI): Calcd For C_11_H_9_O_3_F_2_ : [M+H]^+^: 227.0514 Found: *m/z* 227.0508.

**5-(5-Bromo-2-fluorophenoxy)-3-methylfuran-2(5*H*)-one (2b)**

^1^H NMR (CDCl_3_) δ 2.02 (t, 3H, *J* = 1.5 Hz), 6.25 (t, 1H, *J* = 1.5 Hz), 7.01 (s, 1H), 7.03 (t, 1H, *J* = 1.5 Hz), 7.21 (dt, 3H), 7.58 (d, 1H, *J* = 2.0 Hz), HRMS (ESI): Calcd For C_11_H_11_FBrO_3_ : [M+H]^+^: 286.9714. Found: *m/z* 286.9713.

**5-(5-Bromo-2-chlorophenoxy)-3-methylfuran-2(5*H*)-one (2c)**

^1^H NMR (CDCl_3_) δ 2.04 (d, 3H, *J* = 1.0 Hz), 6.23 (t, 1H, *J* = 1.5 Hz), 7.06 (t, 1H, *J* = 1.5 Hz), 7.21 (dt, 1H, *J* = 8.5, 1.3 Hz), 7.27 (d, 1H, *J* = 8.5 Hz), 7.51 (t, 1H, *J* = 1.8 Hz), HRMS (ESI): Calcd For C_11_H_9_O_3_ClBr : [M+H]^+^: 302.9418 Found: *m/z* 302.9411.

**5-(2-Bromo-5-fluorophenoxy)-3-methylfuran-2(5*H*)-one (2d)**

^1^H NMR (CDCl_3_) δ 2.04 (t, 3H, *J* = 1.8 Hz), 6.23 (t, 1H, *J* = 1.3 Hz), 6.75-6.79 (m, 1H), 7.08 (t, 1H, *J* = 1.5 Hz), 7.11 (dd, 1H, *J* = 9.5, 3.0 Hz), 7.52 (dd, 1H, *J* = 9.0, 1.0 Hz), HRMS (ESI): Calcd For C_11_H_9_O_3_FBr : [M+H]^+^: 286.9714 Found: *m/z* 286.9715.

**5-(5-Bromo-2-cyanophenoxy)-3-methylfuran-2(5*H*)-one (2e)**

^1^H NMR (CDCl_3_) δ 2.06 (t, 3H, *J* = 1.0 Hz), 6.32 (t, 1H, *J* = 1.3 Hz), 7.09 (t, 1H, *J* = 1.5 Hz), 7.34-7.37 (m, 1H), 7.48 (dd, 1H, *J* = 8.5, 0.5 Hz), 7.59 (d, 1H, *J* = 1.5 Hz), HRMS (ESI): Calcd For C_12_H_9_NO_3_Br : [M+H]^+^: 293.9760 Found: *m/z* 293.9756.

**5-(2-Fluoro-5-nitrophenoxy)-3-methylfuran-2(5*H*)-one (2f)**

^1^H NMR (CDCl_3_) δ 2.06 (s, 3H), 6.30 (t, 1H, *J* = 1.3 Hz), 6.95-6.99 (m, 1H), 7.09 (t, 1H, *J* = 1.5 Hz), 7.25 (dd, 1H, *J* = 9.5, 2.5 Hz), 7.99 (dd, 1H, *J* = 9.3, 5.8 Hz), HRMS (ESI): Calcd For C_11_H_9_NO_5_F : [M+H]^+^: 254.0459 Found: *m/z* 254.0459.

**5-(2-Chloro-5-nitrophenoxy)-3-methylfuran-2(5*H*)-one (2g)**

^1^H NMR (CDCl_3_) δ 2.06 (t, 3H, *J* = 1.8 Hz), 6.38 (t, 1H, *J* = 1.5 Hz), 7.10 (t, 1H, *J* = 1.8 Hz), 7.59 (d, 1H, *J* = 8.5 Hz), 7.97 (dd, 1H, *J* = 8.5, 2.5 Hz), 8.21 (d, 1H, *J* = 3.0 Hz), HRMS (ESI): Calcd For C_11_H_9_NO_5_Cl : [M+H]^+^: 270.0164 Found: *m/z* 270.0164.

**5-(2,6-Difluorophenoxy)-3-methylfuran-2(5*H*)-one (3a)**

^1^H NMR (CDCl_3_) δ 1.99 (t, 3H, *J* = 1.5 Hz), 6.28 (d, 1H, *J* = 1.5 Hz), 6.92-6.98 (m, 2H), 7.07 (t, 1H, *J* = 1.8 Hz), 7.08-7.12 (m, 1H), HRMS (ESI): Calcd For C_11_H_9_O_3_F_2_ : [M+H]^+^: 227.0514 Found: *m/z* 227.0514.

**5-(6-Chloro-2-fluorophenoxy)-3-methylfuran-2(5*H*)-one (3b)**

^1^H NMR (CDCl_3_) δ 2.00 (t, 3H, *J* = 1.3 Hz), 6.26 (d, 1H, *J* = 1.3 Hz), 7.04-7.11 (m, 3H), 7.19-7.21 (m, 1H), HRMS (ESI): Calcd For C_11_H_9_O_3_FCl : M^+^: 243.0219 Found: *m/z* 243.0219.

**5-(2,6-dibromophenoxy)-3-methylfuran-2(5*H*)-one (3c)**

^1^H NMR (CDCl_3_) δ 2.04 (d, 3H, *J* = 1.5 Hz), 6.35 (s, 1H), 6.97 (t, 1H, *J* = 8.0 Hz), 7.17 (t, 1H, *J* = 1.3 Hz), 7.57 (d, 2H, *J* = 8.0 Hz), HRMS (ESI): Calcd For C_11_H_9_O_3_Br_2_ : [M+H]^+^: 346.8913 Found: *m/z* 346.8913.

**5-(2-Fluoro-6-nitrophenoxy)-3-methylfuran-2(5*H*)-one (3d)**

^1^H NMR (CDCl_3_) δ 2.02 (t, 3H, *J* = 1.8 Hz), 6.36 (d, 1H, *J* = 1.8 Hz), 7.12 (t, 1H, *J* = 1.8 Hz), 7.32 (td, 1H, *J* = 8.5, 5.0 Hz), 7.45 (td, 1H, *J* = 9.0, 1.5 Hz), 7.73 (dt, 1H, *J* = 8.0, 1.8 Hz),HRMS (ESI): Calcd For C_11_H_9_NO_5_F : [M+H]^+^: 254.0459 Found: *m/z* 254.0455.

**5-(2-Bromo-6-nitrophenoxy)-3-methylfuran-2(5*H*)-one (3e)**

^1^H NMR (CDCl_3_) δ 2.05 (s, 3H), 6.30 (s, 1H), 7.09 (s, 1H), 7.30 (d, 1H, *J* = 9.0 Hz), 7.70 (dd, 1H, *J* = 9.0, 2.5 Hz), 7.73 (d, 1H, *J* = 2.5 Hz), HRMS (ESI): Calcd For C_11_H_9_NO_5_Br : [M+H]^+^: 313.9659 Found: *m/z* 313.9663.

**5-(2-bromo-6-cyanophenoxy)-3-methylfuran-2(5*H*)-one (3f)**

^1^H NMR (CDCl_3_) δ 2.05 (t, 3H, *J* = 1.3 Hz), 6.38 (t, 1H, *J* = 1.5 Hz), 7.19 (t, 1H, *J* = 1.5 Hz), 7.22 (t, 1H, *J* = 8.0 Hz), 7.63 (dd, 1H, *J* = 7.8, 1.3 Hz), 7.86 (dd, 1H, *J* = 8.0, 1.5 Hz), HRMS (ESI): Calcd For C_12_H_9_NO_3_Br : [M+H]^+^: 293.9760 Found: *m/z* 293.9764.

**5-(2,4-Difluorophenoxy)-3-methylfuran-2(5*H*)-one (4a)**

^1^H NMR (CDCl_3_) δ 2.00 (s, 3H), 6.20 (d, 1H, *J* = 1.5 Hz), 6.82-6.92 (m, 2H), 7.02 (s, 1H), 7.27 (m, 1H), HRMS (ESI): Calcd For C_11_H_9_O_3_F_2_ : [M+H]^+^: 227.0514 Found: *m/z* 227.0508.

**5-(4-Chloro-2-fluorophenoxy)-3-methylfuran-2(5*H*)-one (4b)**

^1^H NMR (CDCl_3_) δ 2.01 (s, 3H), 6.23 (s, 1H), 7.02 (s, 1H), 7.10 (dd, 1H, *J* = 8.5, 1.5 Hz), 7.16 (dd, 1H, *J* = 10.0, 2.3 Hz), 7.25 (t, 1H, *J* = 9.3 Hz), HRMS (ESI): Calcd For C_11_H_9_O_3_FCl : [M+H]^+^: 243.0219 Found: *m/z* 243.0217.

**5-(4-Bromo-2-fluorophenoxy)-3-methylfuran-2(5*H*)-one (4c)**

^1^H NMR (CDCl_3_) δ 2.01 (t, 3H, *J* = 1.5 Hz), 6.23 (t, 1H, *J* = 1.5 Hz), 7.03 (t, 1H, *J* = 1.5 Hz), 7.20 (t, 1H, *J* = 8.3 Hz), 7.23-7.26 (m, 1H), 7.30 (dd, 1H, *J* = 9.8, 2.3 Hz), HRMS (ESI): Calcd For C_11_H_9_O_3_FBr : [M+H]^+^: 286.9714 Found: *m/z* 286.9713.

**5-(2-Chloro-4-fluorophenoxy)-3-methylfuran-2(5*H*)-one (4d)**

^1^H NMR (CDCl_3_) δ 2.01 (t, 3H, *J* = 1.5 Hz), 6.17 (t, 1H, *J* = 1.5 Hz), 6.98 (ddd, 1H, *J* = 9.0, 7.5, 3.0 Hz), 7.05 (t, 1H, *J* = 1.5 Hz), 7.16 (dd, 1H, *J* = 8.0, 2.5 Hz), 7.31 (dd, 1H, *J* = 9.3, 5.3 Hz), HRMS (ESI): Calcd For C_11_H_9_O_3_FCl : [M+H]^+^: 243.0219 Found: *m/z* 243.0222.

**5-(4-Bromo-2-chlorophenoxy)-3-methylfuran-2(5*H*)-one (4e)**

^1^H NMR (CDCl_3_) δ 2.02 (t, 3H, *J* = 1.5 Hz), 6.21 (s, 1H), 7.06 (s, 1H), 7.22 (d, 1H, *J* = 8.5 Hz), 7.38 (dd, 1H, *J* = 8.5, 2.0 Hz), 7.56 (d, 1H, *J* = 2.0 Hz), HRMS (ESI): Calcd For C_11_H_9_O_3_ClBr: [M+H]^+^: 302.9418 Found: *m/z* 302.9418.

**5-(2-Bromo-4-fluorophenoxy)-3-methylfuran-2(5*H*)-one (4f)**

^1^H NMR (CDCl_3_) δ 2.02 (d, 3H, *J* = 1.5 Hz), 6.16 (d, 1H, *J* = 1.5 Hz), 7.01-7.05 (m, 1H), 7.07 (t, 1H, *J* = 1.5 Hz), 7.29-7.34 (m, 2H), HRMS (ESI): Calcd For C_11_H_9_O_3_FBr : [M+H]^+^: 286.9714 Found: *m/z* 286.9690.

**5-(2-Bromo-4-chlorophenoxy)-3-methylfuran-2(5*H*)-one (4g)**

^1^H NMR (CDCl_3_) δ 2.03 (s, 3H), 6.19 (d, 1H, *J* = 1.0 Hz), 7.06 (s, 1H), 7.26-7.30 (m, 2H), 7.58 (d, 1H, *J* = 2.0 Hz), HRMS (ESI): Calcd For C_11_H_9_O_3_ClBr: [M+H]^+^: 302.9418 Found: *m/z* 302.9409.

**5-(2,4-Dibromophenoxy)-3-methylfuran-2(5*H*)-one (4h)**

^1^H NMR (CDCl_3_) δ 2.03 (t, 3H, *J* = 1.5 Hz), 6.20 (t, 1H, *J* = 1.5 Hz), 7.06 (t, 1H, *J* = 1.5 Hz), 7.21 (d, 1H, *J* = 8.5 Hz), 7.43 (dd, 1H, *J* = 8.5, 2.5 Hz), 7.72 (d, 1H, *J* = 2.5 Hz), HRMS (ESI): Calcd For C_11_H_9_O_3_Br_2_: [M+H]^+^: 346.8913 Found: *m/z* 346.8892.

**5-(4-Bromo-2-cyanophenoxy)-3-methylfuran-2(5*H*)-one (4i)**

^1^H NMR (CDCl_3_) δ 2.03 (d, 3H, *J* = 0.5 Hz), 6.31 (d, 1H, *J* = 1.0 Hz), 7.15 (d, 1H, *J* = 1.5 Hz), 7.24-7.27 (m, 1H), 7.87-7.90 (m, 2H), HRMS (ESI): Calcd For C_12_H_9_BrNO_3_: [M+H]^+^: 293.9760 Found: *m/z* 293.9759.

**5-(2-Bromo-4-cyanophenoxy)-3-methylfuran-2(5*H*)-one (4j)**

^1^H NMR (CDCl_3_) δ 2.06 (s, 3H), 6.31 (d, 1H, *J* = 1.0 Hz), 7.08 (d, 1H, *J* = 1.5 Hz), 7.40 (d, 1H, *J* = 8.5 Hz), 7.64 (dd, 1H, *J* = 8.5, 1.5 Hz), 7.88 (d, 1H, *J* = 1.5 Hz), HRMS (ESI): Calcd For C_12_H_9_BrNO_3_: [M+H]^+^: 293.9760 Found: *m/z* 293.9760.

**5-(4-Chloro-2-nitrophenoxy)-3-methyl-2(5*H*)-furanone(4k)**

^1^H NMR (CDCl_3_) δ 2.03 (d, 3H, *J* = 2.0 Hz), 6.26 (s, 1H), 7.07 (d, 1H, *J* = 2.0 Hz), 7.47 (d, 1H, *J* = 9.0 Hz), 7.57 (dd, 1H, *J* = 9.0, 2.5 Hz), 7.88 (d, 1H, *J* = 2.5 Hz), HRMS (ESI): Calcd For C_11_H_9_ClNO_5_: [M+H]^+^: 270.0164 Found: *m/z* 270.0164.

**5-(2-Chloro-4-nitrophenoxy)-3-methylfuran-2(5*H*)-one (4l)**

^1^H NMR (CDCl_3_) δ 2.07 (t, 3H, *J* = 1.8 Hz), 6.37 (t, 1H, *J* = 1.5 Hz), 7.10 (t, 1H, *J* = 1.8 Hz), 7.46 (d, 1H, *J* = 9.5 Hz), 8.19 (dd, 1H, *J* = 9.0, 2.5 Hz), 8.33 (d, 1H, *J* = 2.5 Hz), HRMS (ESI): Calcd For C_11_H_9_ClNO_5_: [M+H]^+^: 270.0164 Found: *m/z* 270.0163.
