# Supplementary material for: A Taylor-Made Design of Phenoxyfuranone-Type Strigolactone Mimic
Source: Front Plant Sci. 2017 Jun 20;8:936. doi: 10.3389/fpls.2017.00936 (PMC5477565; doi:10.3389/fpls.2017.00936)
Supplement: Supplementary file 3 [file Image_1.PDF]

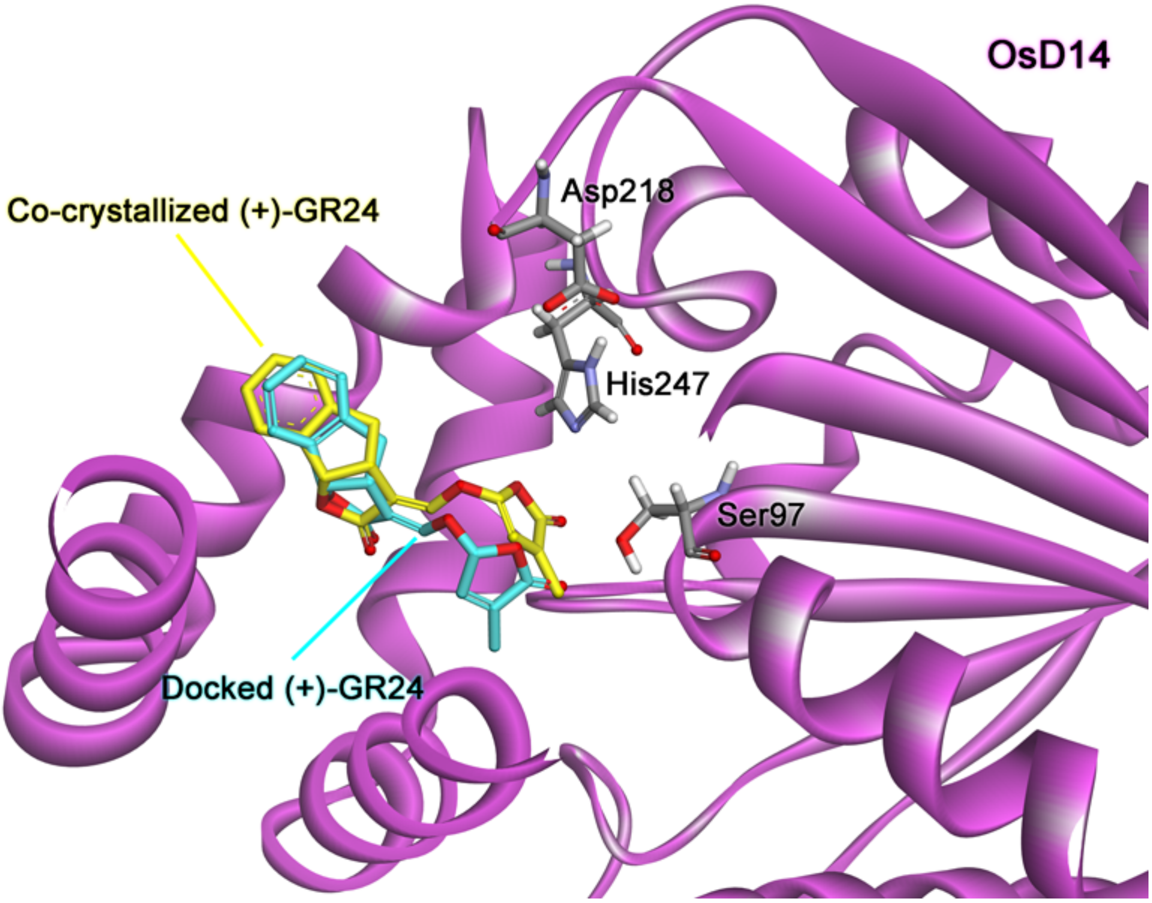

Figure S1. Overlay structure of Docked GR24 on OsD14

Overlay structure of docked (+)-GR24 (cyan stick) on co-crystallised (+)-GR24 (yellow stick) with OsD14 (magenta ribbon). Docked (+)-GR24 was reasonably fitted in SL binding pocket of OsD14.
