# Supplementary material for: A Taylor-Made Design of Phenoxyfuranone-Type Strigolactone Mimic
Source: Front Plant Sci. 2017 Jun 20;8:936. doi: 10.3389/fpls.2017.00936 (PMC5477565; doi:10.3389/fpls.2017.00936)
Supplement: Supplementary file 4 [file Image_2.PDF]

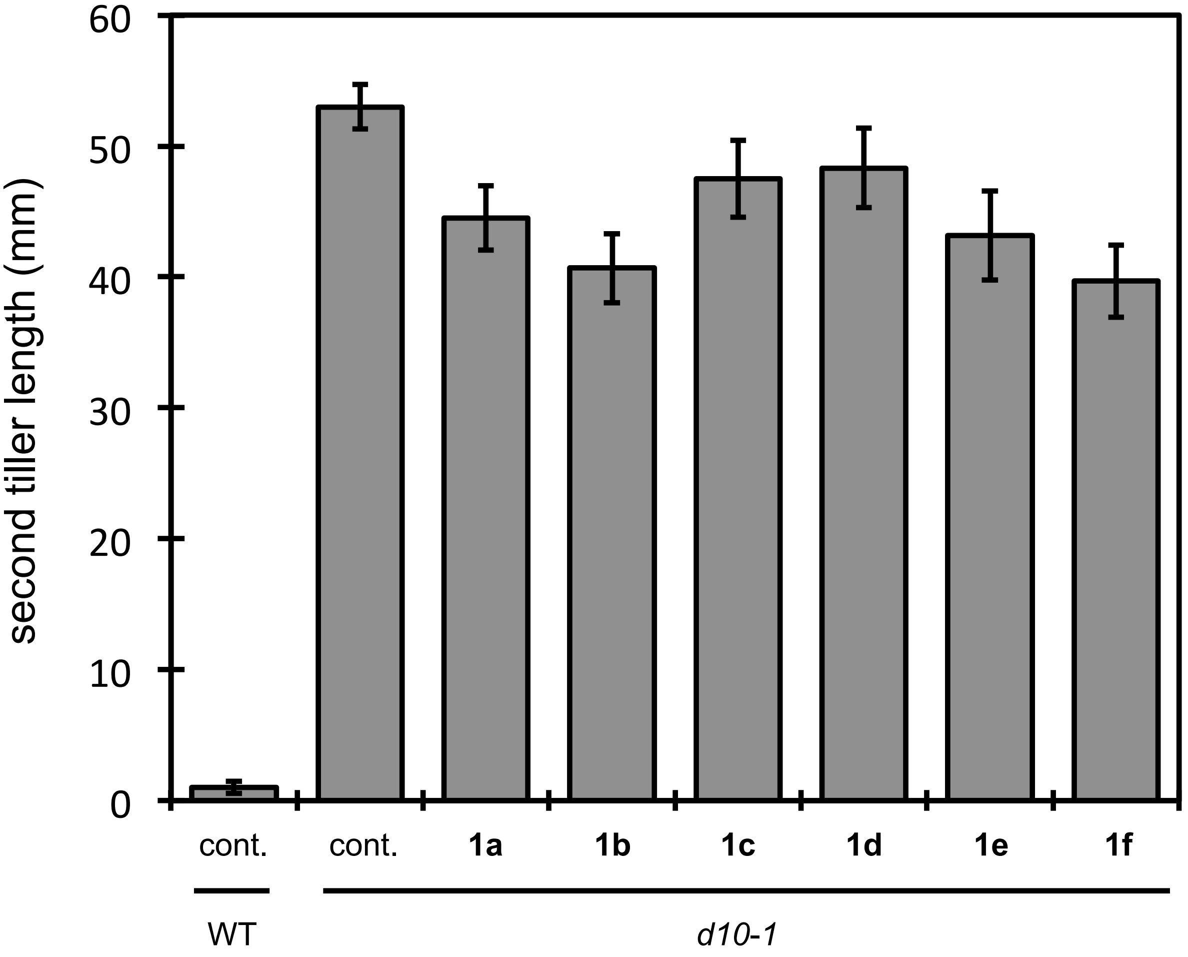

Figure S2. Second tiller length of rice treated with compounds

Each bar indicates average length of second tiller of six rice seedlings 16 days after germination. Error bar means SE. Each seedling was grown in hydroponic culture with each compound at 1 nM concentration for 7 days before measurement (cont. = control).
